# Variation of Natural Streamflow since 1470 in the Middle Yellow River, China

**DOI:** 10.3390/ijerph6112849

**Published:** 2009-11-17

**Authors:** Chi-Yuan Miao, Jin-Ren Ni

**Affiliations:** 1 Department of Environmental Engineering, Peking University, Beijing 100871, China; E-Mail: miaocy@vip.sina.com; 2 The Key Laboratory of Water and Sediment Sciences, Ministry of Education, Beijing 100871, China

**Keywords:** natural streamflow, Yellow River, variation

## Abstract

Nowadays, as the available water resources throughout the World are becoming depleted, in order to manage and plan water resource better, more and more attention is being paid into the fluctuating characteristics of water discharges. However, the preexisting research was mainly focused on the last half century. In this paper, the natural streamflow observed since 1470 at the Sanmenxia station in the middle Yellow River basin was collected, and the methods of variation coefficient, moving average, Mann-Kendall test and wavelet transform were applied to analyze the dynamic characteristics of the streamflow. The results showed that, (1) between 1470 and 2007, the natural streamflow changed 200–919 × 10^8^ m^3^, and water discharge varied moderately; (2) in the middle Yellow River basin, it appears that the most severe and most persistent droughts during *circa* 1868–1990, the periods of 1470s–1490s, 1920s–1930s and 1990s–2000s also presented the condition of sustained low flows; (3) the natural streamflow series shows increasing and decreasing trends during the periods of 1470–1880 and 1881–2007, respectively, but both trends are not significant at >95% confidence; in addition, it is still found the streamflow series shows abrupt changes *circa* 1845, 1935 and 1960, respectively; (4) within a 250-year scale, there are *circa* 11, 26, 67 and 120-year periods for natural streamflow at the Sanmenxia station, and the periodicity of the 120-year one is the strongest. The dynamic characteristics of natural streamflow is the comprehensive result by many influencing factors, such as precipitation, temperature, El Niño-Southern Oscillation, sunspots, human activity, *etc*.

## 1. Introduction

Water is the base of the life on Earth, however, the available water resources throughout the world are becoming depleted, and this problem is aggravated by the increasing population, extending irrigation agriculture and developing industry. Nowadays, it is widely acknowledged that water is a major limiting factor for socio-economic development in the World [1], and water deficiency is stressing the quantity and quality aspects of the natural systems. This has given rise to the problem of optimal management of water resources potential on all parts of the World, more so, in the developing countries like China [2].

River flow plays the central role in the development and management of water resources, but on the other hand, the distribution of streamflows is highly uneven on the temporal scale, so more and more researchers are paying attention to topics in this field, such as streamflow circulation, riverflow oscillation, discharge prediction, *etc*. Williams [3] investigated the nature and causes of cyclical changes in hydrological data of the World, and he detected a correlation between water discharge and sunspots with varying success; Currie [4], Probst and Tardy [5] studied mean annual discharge fluctuation of fifty major rivers distributed around the World, showing that the river flow in North America and Europe fluctuated with the opposite trends, while it presented a synchronous fluctuation in South America and Africa. Whitfield [6] examined the temperature, precipitation and streamflow in British Columbia and the Yukon, finding that streamflow increased throughout the year in the northern region of his study. Woodhouse [7] and Gedalof *et al.* [8] used tree-rings to reconstruct the historical hydrological data in Middle Boulder Creek watershed (Colorado, USA) and Columbia river (Washngton, USA), and then located the drought years over the interannual, decadal, and interdecadal flow regimes. Absalon and Matysik [9] examined the runoff variation in the Upper Oder River basin (Poland), and got the results that the runoffs were in all cases decreasing from 1970 to 2000, but these trends were not significant when using the Student *t* test, in contrast, runoffs increased during 1991–2000 with statistical significance. Sen [10] investigated the riverflow variability in England and Wales by examining the monthly discharge time series from 15 catchments over the period from 1865–2002, and found the discharges exhibited a strong annual cycle.

In China, because the Yangtze and Yellow River are the two largest rivers in China, with a significant domestic status and the number of affected people is multitudinous, and their degraded eco-environments, these rivers have attracted the most attention in China.

In the Yangtze River, it is found that during the past 50 years, although the riverflow patterns were different, no significant trend (at 95% confidence level) was detected for annual water discharges at all hydrologic stations [11], although the natural riverflow increased in summer and decreased greatly in autumn during 1961–2001 [12], and significant positive trends was found in July each year [13]. Zhang *et al.* [14] reported the maximum streamflow and annual maximum water level change at three major stations of the Yangtze River during the past 130 years, and the results showed there was a significant upward trend in streamflow in the middle Yangtze River. In the Yellow River, although the water discharges changed on the interannual and longer time scales [15,16], no significant trend was detected from 1956–2001 [17]. Analyzed further, the river flow in the middle Yellow River [18], lower Yellow River [19] and the water fluxes to the sea [20] have declined significantly since the 1970s.

The preexisting researches were mainly focused on the last half century; but statistical analysis of the riverflow variation depends on the availability of long time series, as these include richer information. However, systematic measurement of discharges in the modern era started relatively late, and it is relatively difficult to collect the historical data over centuries. The objectives in this paper is, to analyze the oscillating character and change cycles in the middle Yellow River basin, based on the reconstructed historical data (1470–2007) from the Sanmenxia station, and discuss the influencing factors. The study will be helpful for policy-makers to manage the water resource more effectively.

## 2. Materials and Methods

### 2.1. Study Area

**Figure 1.**
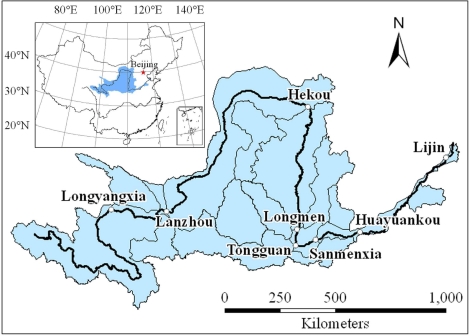
Location of Sanmenxia station in the Yellow River basin.

The Yellow River (Huanghe) is the second largest river in China, with a total length of 5,464 km. It originates in the northeast of the Tibetan Plateau, runs across the Loess Plateau of North China and the Ordos Plateau, and flows eastwards to the Bohai Sea, via semi-arid and semi-humid regions (Figure 1). The Yellow River basin (7.52 × 10^5^ km^2^) is one of the most important drainage areas in China, directly supporting a population of some 110 million people, mostly farmers and rural residents. The Yellow River basin covers nine provinces/autonomous regions, *i.e.*, Qinghai, Sichuang, Gansu, Ningxia, Inner Mongolia, Shaanxi, Shanxi, Henan and Shandong. On average, the Yellow River had 58 billion m^3^ of runoff (during the period of 1919–1975), accounting for 2% of the total runoff of China [21].

Since the late part of the last century, the middle and lower reaches of the Yellow River have periodically suffered from floods or droughts, which consequently have led to serious conflicts between ecological water supply and demand, and decreased the health level of the Yellow River ecosystem [22]. The Sanmenxia gauging station, located at the outlet of the Sanmenxia reservoir, is one of the major hydrological control stations in the main channel of the Yellow River (Figure 1), and the drainage area upper the Sanmenxia station occupies the 91.9% area of the Yellow River basin. Due to frequent flood/drought event occurrences, the Sanmenxia station has been used as a hydrological monitoring station for a long time in history.

### 2.2. Data Collect

In the Yellow River basin, though the earliest official survey of water discharge started in 1919, some flood/drought events have been fortunately recorded in the local history logs. Wang *et al.* [23], based on the flood alarm of the Yellow River in Qing Dynasty and the drought-flood distribution of China in the recent 500 years [24], reconstructed the natural streamflow of Sanmenxia station during 1470–1918. The annual streamflow at the Sanmenxia station during 1919–1997 was observed by the hydrological bureau, Yellow River Conservancy Commission (YRCC), and the natural streamflow data during 1998–2007 was collected from the water resource communique, issued by the Ministry of Water Resources (MWR).

### 2.3. Analysis Methods

#### (1) Statistical analysis

One-Sample Kolmogorov-Smirnov Test was conducted to detect the normal distribution. In order to detect the water discharge oscillation on the decadal scale, the 11-year moving average was used, and the average value among 11 years was placed into the 6th year position. In addition, the variation coefficient *Cv* were adopted to reflect the dispersion variation degree of natural streamflow:

(1) $$Cv\;=\;\frac{\sqrt{\frac{1}{n-1}\sum_{i=1}^{n}{\left(x_{i}\;-\;\bar{x}\right)}^{2}}}{\bar{x}}$$

where, *n* is the length of the streamflow series, *x_i_* is the natural streamflow in the *i*th year, *x̄* is the multi-year average value of streamflow, and *t̄* = (*n* +1)/2. It is generally believed that *Cv* < 0.1 means weak variability, 0.1 ≤ *Cv* ≤ 1 means moderate variability, and *Cv* > 1 means strong variability. The statistical analysis was performed using Excel 2003 and Spss 13.0.

#### (2) Low flow analysis

In order to evaluate the persistence of drought over the period of 1470–2007, the streamflow series was de-noised using a range of multiyear center moving averages. According the references from Woodhous [7] and Gedalof *et al.* [8], window lengths of 1, 5, 11 and 25 years were chosen to characterize interannual, decadal, and interdecadal flow regimes. Years that fell into the lowest 15% (*i.e.*, the 80 lowest flow years in this research) were collected and ranked, and then plotted as a function of time.

#### (3) Mann-Kendall test

The rank-based Mann-Kendall method (MK), put forward by Mann and Kendall [25,26], is a nonparametric and commonly used method to assess the significance of monotonic trends in hydro-meteorological time series (e.g., [11,14]). This test has the advantage of not assuming any distribution form for the data and has similar power as its parametric competitors [27]. Therefore, it is highly recommended for general use by the World Meteorological Organization [28]. In this study, the Mann-Kendall test procedure follows Gerstengarbe and Werner [29] who used the method to test an assumption about the beginning of the development of trend within a sample (*x*_1_, *x*_2_, …, *x*_n_) of the random variable *x*, based on the rank series r of the progressive and retrograde rows of this sample. The assumption (null hypothesis) is formulated as follows: the sample under investigation shows no beginning of a developing trend. The following test is done to prove or to disprove the assumption, first a MK test statistic, *d_k_* is calculated:

(2) $$d_{k}\;=\;\sum_{i=1}^{k}r_{i}(2\;\leq \;k\;\leq \;n)$$

where:

(3) $$r_{i}\;=\;\{\begin{array}{ll} +1 & \text{if}\;x_{i}\;>\;x_{j}\\ 0 & \text{otherwise} \end{array}\;\;\;\;(j=1,\;2,\;\ldots ,\;i)$$

Under the null hypothesis of no trend, the statistic *d_k_* is distributed as a normal distribution with the expected value of *E*[*d_k_*] and the variance *Var*[*d_k_*] as follows:

(4) $$E[d_{k}]\;=\;\frac{n(n-1)}{4}$$

(5) $$Var[d_{k}]\;=\;\frac{n(n-1)(2n\;+\;5)}{72}$$

Under the above assumption, the definition of the statistic index Zk is calculated as:

(6) $$Z_{k}\;=\;\frac{d_{k}\;-\;E[d_{k}]}{\sqrt{Var[d_{k}]}}\;\;\;\;\;\;\;\;\;\;\;(k\;=\;1,\;2,\;3,\;\ldots ,\;n)$$

In a two-sided test for trend, the null hypothesis is rejected at the significance level of α if |*Z*| > *Z*_(1−α/2)_, where *Z*_(1−α/2)_ is the critical value of the standard normal distribution with a probability exceeding α/2. A positive *Z* value denotes a positive trend and a negative *Z* value denotes a negative trend. In this paper, the significant level α = 5% is used. In contrast to the traditional MK test which calculates above statistic variables only once for the whole sample, the corresponding rank series for so-called retrograde rows are similarly obtained for the retrograde sample (*x*_n_, *x*_n−1_, … , *x*_1_). Following the same procedure as shown in Equations 2–6, the statistic variables, *d_k_*, *E*[*d_k_*], *Var*[*d_k_*] and *Z*_k_ will be calculated for the retrograde sample. The *Z* values calculated with progressive and retrograde series are named *Z*_1_ and *Z*_2_, respectively in this paper. The intersection point of the two lines, *Z*_1_ and *Z*_2_ (k = 1, 2, …, n) locates the time of abrupt change for time series, if the intersection point is between the two confidence lines. The null hypothesis must be rejected if the intersection point is significant at 5% significant level (*i.e.*, outside the 95% confidence interval). The Mann-Kendall calculation was accomplished with Matlab 7.0.

#### (4) Wavelet analysis

The basic objective of the wavelet transform is to achieve a complete time-scale (or shift-scale) representation of localized and transient phenomena occurring at different time scales. Based on the results of time-scale distribution, it is easy to analyze the periodicity of streamflow series. For time series *f* (*t*) ∈ *L*^2^ (*R*), the continuous wavelet transform (CWT) is defined as the sum over all time of the real signal *f*(*t*) multiplied by the scalded [30], shifted versions of the wavelet function *ψ, i.e.*,:

(7) $$W_{f}(a,\;b)\;=\;{\left|a\right|}^{\frac{-1}{2}}\int_{-\infty }^{+\infty }f(t)\Psi (\frac{t-b}{a})dt$$

where the wavelet coefficients *W_f_*(*a*,*b*) are the result of the CWT of signal *f*(*t*), *a* and *b* is the scale and the translation, respectively.

The key point of wavelet transform lies in the selection of wavelet function. The real part and imaginary part of complex wavelet has a phase difference of π/2, which can eliminate the modular vibration of wavelet transform coefficient of real form, so in this study, the Complex Morlet wavelet was adopted to analyze the periodic characteristics of natural streamflow in relation to time. The Complex Morlet wavelet is a single-frequency complex sinusoidal function tapered with a Gaussian window, and is expressed as:

(8) $$\psi (t)\;=\;e^{ict}e^{-t^{2}/2}$$

where, *c* is a constant; *i* represents imaginary part. The main period of on time series is obtained by wavelet variance [21], which is expressed as follows:

(9) $$Var(a)\;=\;\sum {\left(W_{f}\right)}^{2}(a,\;b)$$

where *Var*(*a*) is the wavelet variance. Since wavelet variance denotes the distribution of wavelet energy by scale (period), the domain predominant periods of one time series can obtained from its extreme values. All the wavelet transform processes were carried out with the Matlab 7.0 software.

### 2.4. Data Preliminary Analysis

Mann-Kendall assumed that sample data are serially independent, so a series with a positive serial correlation will inflate the variance of the estimated mean, and hence the effective sample size contains less information about the mean than a random series [31], consequently this increases the possibility of rejecting the null hypothesis (type I error) [32,33]. The preliminary analysis for checking serial correlation was examined by Equation 10:

(10) $$r_{k}\;=\;\frac{\sum_{i=1}^{n-k}\left(x_{i}\;-\;\bar{x})\;(x_{i+k}\;-\;\bar{x}\right)}{\sum_{i=1}^{n}{\left(x_{i}\;-\;\bar{x}\right)}^{2}}$$

where *n* is the length of the streamflow series, *x_i_* is the natural streamflow in the *i*th year, $\bar{x}\;=\;\sum_{t=1}^{n}x_{t}$ is the overall mean, *r_k_* is the autocorrelation coefficient at lag *k*.

It follows that the critical level of correlation for 95% significance is $-1/n\;\pm \;2/\sqrt{n}$ (two sided), and are often further approximated $r_{.95}\;≅\;0\;\pm \;2/\sqrt{n}$ (two sides) for the large time series [34]. If the autocorrelation coefficient at a certain order is outside the confidence interval (e.g., 95% confidence level), which indicates the time series is autocorrelation and not independent at this order (reject the null hypothesis).

**Figure 2.**
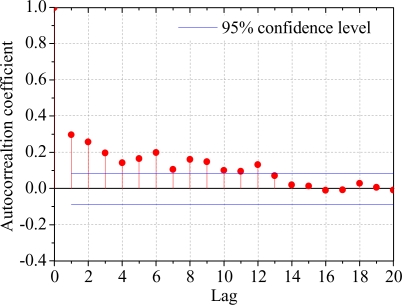
Autocorrelation results.

The results of serial correlation analysis indicated the natural streamflow at the Sanmenxia station had significant autocorrelation at the lag = 12 (Figure 2). So before the MK test, it was necessary to eliminate the autocorrelation of the series. The MK-TRPW procedure was used to limit the influence of serial correlation, and the autoregressive model AR(12) was applied to filter the autocorrelation components. The MK-TRPW processes mainly includes four steps: (1) detrending the time series, and dividing the original series into trend series and residual series; (2) calculating the parameters of autoregressive (AR) model for the residual series; (3) removing AR component and (4) combining the series after removing AR component and trend series. For more detailed procedures see reference [33].

## 3. Results

### 3.1. Statistical Characteristics

**Figure 3.**
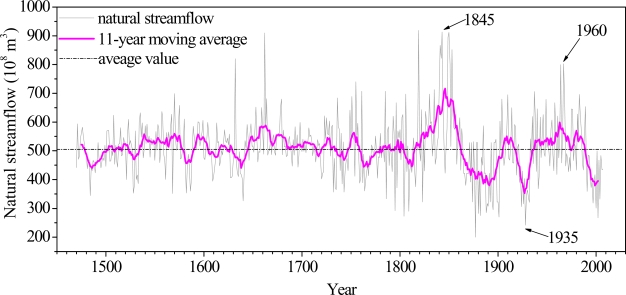
Annual natural streamflow at the Sanmenxia station.

The result from the One-Sample Kolmogorov-Smirnov Test rejects the null hypothesis of normal distribution (*P* = 0.019, <0.05), which explains the diversion and change of natural streamflow is consistent with the natural laws: natural streamflow is the result of comprehensive effect by different factors. The streamflow series during 1470–2007 can be divided into two periods approximately: 1470–1810 and 1811–2007, according to the 11-year moving average (Figure 3).

The variation coefficient *Cv* of natural streamflow between 1470–2007 is 0.2023, which means there was moderate variability over the whole period. Calculated separately, the variation coefficients of natural streamflow during 1470–1810 and 1811–2007 are 0.1576 and 0.2640 respectively, the variable degree of streamflow series during 1811–2007 is increased further, which is in accord with Figure 3.

**Figure 4.**
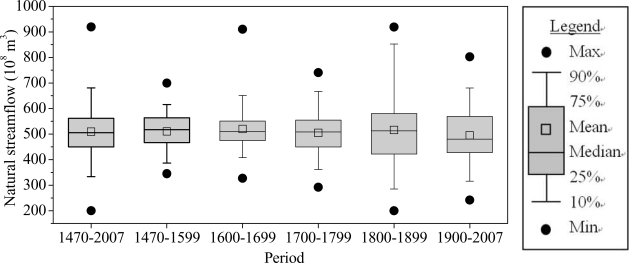
Distribution of natural streamflow at the Sanmenxia station.

The natural streamflow amount is concentrated 200–919 × 10^8^ m^3^ during 1470–2007, the mean value is 510.37 × 10^8^ m^3^ (Figure 4), and although the distribution of natural streamflow in different centuries are a little different, the mean and median is close among the different periods.

### 3.2. Drought Events

**Figure 5.**
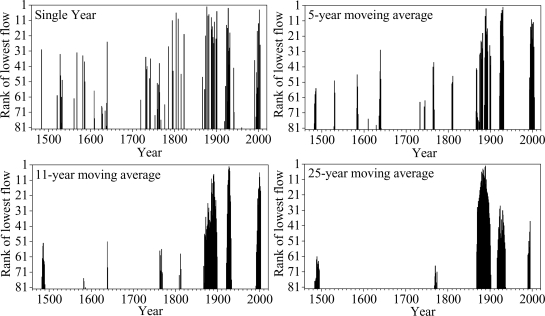
Distribution of natural streamflow for the lowest 15th percentile over the period 1470–2007. Low rankings are indicated by the bars with different height, longer bar represents lower flow event.

According to the evaluated criterion, the distribution of single-year low flow events is fairly frequent over time (Figure 5), although there is a conspicuous cluster of low flow years during 1870s–1900s, 1920s–1930s, and 1990s–2000s.

Intervals of persistent drought become more evident when longer windows lengths are considered. In particular, the interval from circa 1868–1900 appears to be the most severe and most persistent drought on record (25-year windows). The 1470s–1490s, 1920s–1930s and 1990s–2000s also emerge as periods of sustained low flows. Several of the low flow events from the late 19^th^ century, such as 1877, 1928, 1901 and 2002 (several longest bar in Figure 5), are notable because they are more extreme than any that have occurred before this period. The final drought event appeared at the end of 20th century, and their relatively frequent and continual occurrence, may have implication for the future water resource planning. The periods circa 1640–1720, 1822–1865 and 1945–1990 are notable for having no multiyear droughts in the bottom 15th percentile.

### 3.3. Mann-Kendall Test

**Figure 6.**
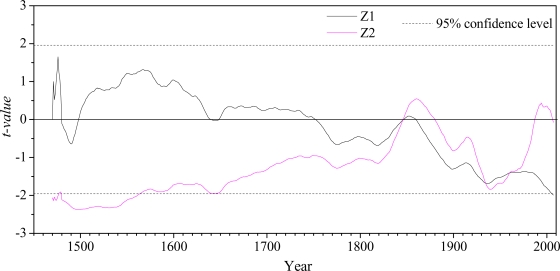
Result of Mann-Kendall test.

The results of the Mann-Kendall test are shown in Figure 6. It can be seen that during 1470–1880, most of the natural streamflow shows an increasing trend (Z_1_ > 0), but the trend is not significant at >95% confidence level; and after 1880 the natural streamflow presents a decreasing trend (Z_1_ < 0), and the trend is still not significant at >95% confidence. For the streamflow series at the Sanmenxia station, the intersection point of the Z_1_ and Z_2_ curves occurs at *circa* 1845, 1935 and 1960, respectively. According to the Mann-Kendall method, the intersection point of *Z*_1_ and *Z*_2_ gives the point of jumping change, which illuminates several abrupt points appeared during 14707–2007, and the result is consistent with Figure 3. Thus it is confirmed that the Mann-Kendall test is an effective tool to detect abrupt changes, even when multiple jumping points exists.

### 3.4. Wavelet Transform

In order to reveal the long-term changes rather than rapid changes in the natural streamflow series, a longer time scale (250-year) is chosen. The wavelet coefficient contour map of the natural streamflow series is plotted based on the above method of Morlet wavelet transform. The intensity at each *x-y* point represents the magnitude of the wavelet coefficient includes both the intensity and the phase of the signal variation, at particular scales and locations in wave domain (the time-frequency domain). For the streamflow wavelet coefficient, when the real part coefficient is a positive value, it means that the streamflow quantity is higher, and vice versa. From the real part periodic change, the variation structures with higher flow and lower flow phases are clearly shown within the different time scales. The lower scale changes are more complex and be comprised by the higher scales.

**Figure 7.**
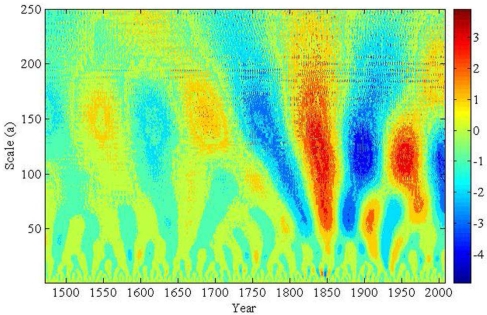
Real part wavelet coefficient contour map of natural streamflow.

When considering the natural streamflow at the Sanmenxia station, the real part of the wavelet transform is shown in Figure 7. In the figure, the red regions represent more abundant streamflow, blue regions mean lower and other colors show the middle amounts. It is seen that the streamflow has 100–150, 50–75, 20–30 and about 10-year periodical character within the 250-year scale. For the 100–150 year scale, there are *circa* four-cycle oscillations, and the oscillation cycle exists the decreasing trend from 150-year to 100-year after 1740. The periods of 1515–1585, 1650–1725, 1800–1875 and 1930–1980 are the abundant-water period, while 1470–1514, 1586–1649, 1726–1799 and 1876–1929 are the low-water period. The streamflow series would be a state of low-water period after 1980.

For the 50–75 scale, there are more than eight-cycle oscillations. The periods of 1470–1490, 1530–1570, 1595–1625, 1650–1680, 1725–1750, 1775–1800, 1830–1865, 1890–1925, 1960–1980 are the abundant-water periods, while 1491–1529, 1571–1594, 1626–1649, 1681–1724, 1751–1774, 1801–1829, 1866–1889, 1926–1959 and after 1980 are the low-water periods. For the 20–30 and 10-year scales, there are more cycle oscillations, and the oscillation frequencies of annual natural streamflow are higher and more complicated.

**Figure 8.**
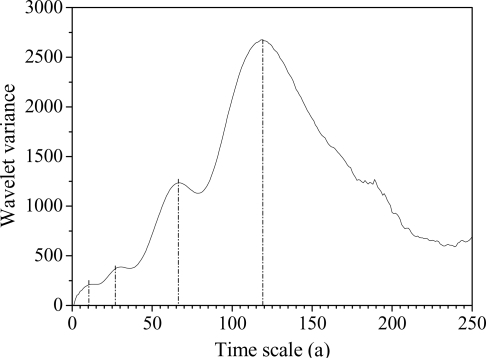
Wavelet variance figure of Morlet wavelet transform coefficients.

The wavelet variance of the streamflow at the Sanmenxia station is computed based on Equation 10. The main period can be obtained from the peak values of variance. Within a 250-year scale, there are circa 11, 26, 67 and 120-year periods, and the 120-year period is the strongest (Figure 8).

## 4. Discussion

**Figure 9.**
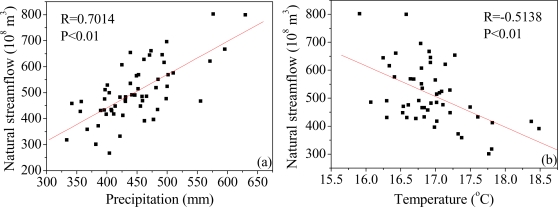
Correlation between natural streamflow and precipitation (a), temperature (b). The annual streamflow observes at the Sanmenxia station, the annual precipitation and temperature is the annual average value in the whole Yellow River basin.

Natural streamflow is the result by hydrological cycles of precipitation, infiltration, evapotranspiration and other components, which leads to the natural water discharge with periodicity and hydrological anomaly. Precipitation is the direct source of natural runoff, and provides the material source for water discharges. Due to the lack of corresponding historical precipitation records, and considering the basin upper Sanmenxia occupies more than 90% area of Yellow River basin, the correlation between natural streamflow (in Sanmenxia station) and precipitation (in Yellow River basin) during 1950–2007 was analyzed (Figure 9a). The significant correlation (R = 0.7014, P < 0.01) between precipitation and natural streamflow exhibits the direct effect of precipitation on natural streamflow. As long as the rainfall intensity is higher than the infiltration rate of underlying surface, or the precipitation exceed the land’s storage capacity, runoff will be generated. Even if the above mentioned event cannot happen, precipitation still can indirectly affect the runoff for the next time by modifying the water content of the ground, *etc*.

Temperature is another critical factor for natural water discharge, and the effect of temperature rises on water discharge is mainly evident in three aspects: the first is to increase potential and actual evaporation, which is unfavorable for discharge yield; the second is beneficial for the thawing of ice and snow, which will increases runoff in the short term; the third is to change the form of precipitation (temperature rising can change snowfall into rainfall) and then change the conditions for runoff generation. The negative correlation (P < 0.01) between natural streamflow and annual average temperature (in the whole Yellow River) was found in this research (Figure 9b). Beside the direct temperature record, the ice-snow amount at the South Pole is another indirect index to measure the temperature conditions. Wang and Liu [35] analyzed the correlation between the amount of ice-snow of the South Pole and the amount of annual natural streamflow at Sanmenxia Station since 1764, it is found that there was a notable reverse correlation between them, with the main wet and dry periods corresponding to the periods of less and more ice and snow at the South Pole, respectively.

**Figure 10.**
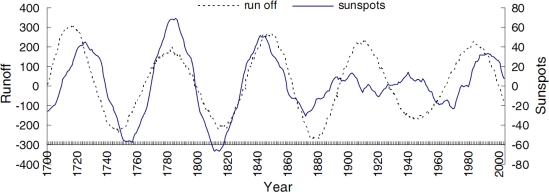
Wavelet coefficient changes of natural streamflow at the Sanmenxia station and sunspots from 1700 to 2003 on a 60-year scale (cited from [21]).

In addition, El Niño-Southern Oscillation (ENSO) and sunspots are undeniable influencing factors, their effects be implemented mostly through their effect on climatic and hydrological cycles. Mechoso and Iribarren [36] have found a relationship between the ENSO phase and streamflow in the Negro and Uruguay Rivers. The same result was found by Xu *et al.* [37], Gong and Wang [38]. Li *et al.* [21] found a correlation between sunspots and natural streamflow when considering a 60-year scale, and the correlation coefficient is 0.65 (Figure 10).

Besides the above natural conditions, human activity is another influencing factor. However, when the natural streamflow is calculated, the water-consumed by human being, such as irrigation water, industrial water and domestic water, *etc*. had already be compensated for, so the man-made influence on natural streamflow is weakened. Most of influence from human beings is presented indirectly through influence on the climatic surroundings and underlying surface conditions, *etc*.

## 5. Conclusions

Natural streamflow is influenced by precipitation, temperature, ENSO, sunspots, human activities, *etc*., which results in anomalous changes and unobvious trends for the natural streamflows. Based on the historical data since 1470, the natural streamflow at the Sanmenxia station in Yellow River basin was analyzed.

As can be concluded, the natural streamflow at the Sanmenxia station presents moderate variability during the whole period 1470–2007, and it appears that some persistent drought intervals exist, when longer windows lengths, such as 1868–1900, 1470s–1490s, 1920s–1930s and 1990s–2000 are considered. By use of the Mann-Kendall test, it is found the natural streamflow during 1470–1880 and 1880–2007 present increasing and decreasing trends, respectively, however both trends are not significant at >95% confidence level. It is still found the streamflow series shoes an abrupt change *circa* 1845, 1935 and 1960, respectively. Through the wavelet transform, it is detected that within the 250-year scale, there are 11, 26, 67 and 120-year periods for the natural streamflow at the Sanmenxia station, and the periodicity of 120-year is the strongest.
